# Editorial: Cancer metastasis through the lymphovascular system: molecular mechanisms of cancer metastasis

**DOI:** 10.1007/s10585-024-10272-8

**Published:** 2024-02-28

**Authors:** Stanley P. Leong, S. David Nathanson, Jonathan S. Zager

**Affiliations:** 1 California Pacific Medical Center and Research Institute, San Francisco, CA USA; 2 University of California San Francisco School of Medicine, San Francisco, CA USA; 3 Department of Surgery, Henry Ford Health, 2799 W. Grand Blvd, Detroit, MI 48202 USA; 4 Cancer Center, Henry Ford Health, Detroit, MI USA; 5 Department of Cutaneous Oncology, Moffitt Cancer Center, Tampa, FL USA; 6 Department of Oncologic Sciences, University of South Florida Morsani College of Medicine, Tampa, FL USA

**Keywords:** Lymphovascular, Cancer metastasis, Molecular mechanisms

The International Congress on Cancer Metastasis through the Lymphovascular System was first held in 2005, with numerous publications summarizing these biennial meetings. In 2022, we summarized these significant findings in our recently published book on Cancer Metastasis through the Lymphovascular System [1]. Cancer heterogeneity due to mutation [2, 3] and epigenetic changes [4] with association of formation of neoantigens [5, 6] is well known. Cancer progression relating to the cancer microenvironment and the mechanisms of cancer metastasis are under intense study. The 9th International Congress on Cancer Metastasis through the Lymphovascular System, being held between May 4–6, 2019, in San Francisco (www.cancermetastasisconference.org), addressed the molecular mechanisms of cancer metastasis from the cancer microenvironment to the sentinel lymph nodes in the majority of the cases, then to systemic sites in 21 review articles and 2 commentaries.

In this Special Issue: Molecular Mechanisms of Cancer Metastasis [7], Carolina Rodriquez-Tirado, and Soledad Sosa address the origins and behavior of disseminated cancer cells during minimal residual disease and dormancy.

Jamie Magrill, Dan Moldoveanu, and Ian Watson attempt to map the spatial immune landscapes of the melanoma microenvironment. Likewise, Wendi Liu, Anusha Puri, Doris Fu, Lee Chan, Cassia Wang, Manolis Kellis, and Jiekun Yang dissect the cancer microenvironment in response to immune checkpoint inhibitors via single-cell and spatial transcriptomics. Further, Cassia Wang, Lee Chen, Doris Fu, Wendi Liu, Anusha Puri, Manolis Kellis, and Jiekun Yang define the antigen-presenting cells in cancer immunity relating to the mediation of immune checkpoint blockade.

Regarding the host immune system, Pamela Basto and Nathan Reticker-Flynn interrogate the role of lymph node metastasis in systemic immune surveillance. David Jackson describes the trafficking of immune cells and potentially that of cancer cells through the lymphatic system. Stanley Leong and Marlys Witte address the route of cancer metastasis through the lymphatic versus blood vessels. Circulating cancer cells can be detected by ‘liquid biopsy’ of cancer. Daniel Smit, Svenja Schneegans, and Klaus Pantel summarize the clinical applications of circulating cancer cells in patients with solid tumors. As a commentary, Tetiana Bowley and Dario Marchetti have applied liquid biopsy to identify the RPL/RPS gene signature of melanoma circulating tumor cells to be associated with brain metastasis.

Regarding resistance to cancer treatment, Abhilash Deo, Jonathan Sleeman, and Yuval Shaked show that the development of resistance to cancer treatment may result in recurrence and metastasis. Using the model of bromodomain inhibition, Imran Khan and Mohammed Kashani-Sabet demonstrate that such inhibition may overcome treatment resistance in melanoma.

As sentinel lymph node biopsy has become a significant staging procedure to define the status of the regional lymph nodes, especially in melanoma and breast cancer, several papers are devoted to the biological and clinical significance of sentinel lymph nodes. David Nathanson and Ian Wood deliver the Donald L Morton Memorial Lecture from a personal perspective as a commentary. Jonathan Zager and David Hyams describe the potential utility of 31 gene analyses of melanoma to help guide treatment and surveillance of melanoma. Mark Faries emphasizes the critical role of melanoma sentinel lymph nodes for optimal treatment. In addition, Stanley Leong and Marlys Witte, in the paper mentioned above, re-introduce the concept of the development of a pre-metastatic niche in the sentinel lymph nodes by cytokines released by primary cancer so that the sentinel lymph nodes become pre-conditioned to accept cancer cells to grow and proliferate. Theresa Schwartz summarizes the role of regional lymph nodes in breast cancer and specifies the need for sentinel lymph node biopsy to stage the axilla for breast cancer.

In the Henry Ford Health Symposium: Molecular Mechanisms of Breast Cancer Metastasis organized by David Nathanson, several submitted contributions include: (1) Multifaceted roles of lymphatic endothelial cells by Lothar Dieterich and Emma Reynaud; (2) The evolving cancer niche interaction during bone colonization by Xian Zhang and Yi-Hsuan Wu; (3) Anatomic and molecular characteristics associated with breast cancer metastasis by Dhananjay Chitale; and (4) Can we cure metastatic breast cancer? by Lajos Pusztai and Alejandro Rios-Hoyo.

The section on Novel Frontiers in the Treatment of Metastatic Melanoma includes the following papers: (1) Neoadjuvant therapy for advanced metastatic melanoma nodal disease by Sharon Cimarron and Giorgos Karakousis; (2) Updates on intralesional monotherapy and combination therapy for in-transit metastases from metastatic melanoma by Danielle DePalo, Matthew Perez, Anne Hulbers, Roger Bagge and Jonathan Zager; and (3) Current status on regional perfusions for in-transit lymphatic metastasis of the limb and uveal melanoma metastatic to the liver by Anne Huibers, Danielle DePalo, Matthew Perez, Jonathan Zager and Roger Bagge.

The paper on Immune Responses against Cancer by Stanley Leong describes the background of immune interaction with cancer, summary of the checkpoint inhibition immunotherapy trials and the diversity of T cell repertoire against cancer. Kevin Kim summarizes the personalized therapy in oncology: melanoma as a model for the paradigm of molecular-targeted treatment approaches. Jessica Xing and Dino Stea delve into the molecular mechanisms of sensitivity and resistance to radiotherapy.

Finally, Michael Bernas, Sara Al-Ghadban, Saskia Thiadens, Karen Ashforth, Walter Lin, Bauback Safa, Rudolf Buntic, Michael Paukshto, Alexandra Rovnaya, and Margaret McNeely describe the etiology and treatment of cancer-related secondary lymphedema, which is associated with cancer treatment including surgery and radiation therapy.

The above-described manuscripts have been separately published by Clinical and Experimental Metastasis and indexed in Pubmed. The entire set of these publications based on the plenary presentations of the 9th International Congress on Cancer Metastasis through the Lymphovascular System held between May 4–6, 2019, in San Francisco (www.cancermetastasisconference.org) have been compiled into this Special Issue in print format as Cancer Metastasis through the Lymphovascular System: Molecular Mechanisms of Metastasis to be published by Clinical and Experimental Metastasis of Springer Nature.

We greatly appreciate the excellent presentations of our speakers, and more so, they have graciously responded to our request to summarize their presentations in review articles or commentaries to be published in this Special Issue. We are grateful to the sponsors and exhibitors who have made this Congress successful. They are acknowledged on our website (www.cancermetastasisconference.org). We are indebted to the Editorial Board of the Clinical and Experimental Metastasis for allowing and helping us to publish the 9th International Congress on Cancer Metastasis presentations in this Special Issue. We plan to launch the next 10th International Congress on Cancer Metastasis through the Lymphovascular System from October 29–31, 2025 in San Francisco.

## Data Availability

No datasets were generated or analysed during the current study.

## References

[1] Leong SP, Nathanson SD, Zager JS (eds) (2022) *Cancer Metastasis Through the Lymphovascular System*, Springer Nature

[2] Steuer CE, Ramalingam SS (2018) Tumor Mutation Burden: leading immunotherapy to the era of Precision Medicine? J Clin Oncol 36(7):631–632. 10.1200/JCO.2017.76.8770 29337637 10.1200/JCO.2017.76.8770

[3] Simpson D, Ferguson R, Martinez CN, Kazlow E, Moran U, Heguy A et al (2017) Mutation burden as a potential prognostic marker of melanoma progression and survival. J Clin Oncol 35(15suppl):9567–9567. 10.1200/JCO.2017.35.15_suppl.9567 10.1200/JCO.2017.35.15_suppl.9567

[4] Iacobuzio-Donahue CA (2009) Epigenetic changes in Cancer. Annu Rev Pathol 4(1):229–249. 10.1146/annurev.pathol.3.121806.151442 18840073 10.1146/annurev.pathol.3.121806.151442

[5] Leong SP, Ballesteros-Merino C, Jensen SM, Marwitz S, Bifulco C, Fox BA, Skoberne M (2018) Novel frontiers in detecting cancer metastasis. Clin Exp Metastasis 35(5–6):403–412. 10.1007/s10585-018-9918-6 30022365 10.1007/s10585-018-9918-6

[6] Ma W, Pham B, Li T (2022) Cancer neoantigens as potential targets for immunotherapy. Clin Exp Metastasis 39(1):51–60. 10.1007/s10585-021-10091-1 33950415 10.1007/s10585-021-10091-1 PMC8097110

[7] Special Issue (by invitation only) : Cancer Metastasis through the Lymphovascular System: Molecular Mechanisms of Cancer Metastasis (2023). *Clin Exp Metastasis*

