# Correction to: Editorial: Cancer metastasis through the lymphovascular system: molecular mechanisms of cancer metastasis

**DOI:** 10.1007/s10585-024-10287-1

**Published:** 2024-05-22

**Authors:** Stanley P. Leong, S. David Nathanson, Jonathan S. Zager

**Affiliations:** 1 California Pacific Medical Center and Research Institute, San Francisco, CA USA; 2 University of California San Francisco School of Medicine, San Francisco, CA USA; 3 Department of Surgery, Henry Ford Health, 2799 W. Grand Blvd, Detroit, MI 48202 USA; 4 Cancer Center, Henry Ford Health, Detroit, MI USA; 5 Department of Cutaneous Oncology, Moffitt Cancer Center, Tampa, FL USA; 6 Department of Oncologic Sciences, University of South Florida Morsani College of Medicine, Tampa, FL USA

**Clinical & Experimental Metastasis**

10.1007/s10585-024-10272-8.

In the sentence beginning ‘The 9th International Congress…’ in this article, the text. ‘The 9th International Congress on Cancer Metastasis through the Lymphovascular System, being held between May 4–6, 2019, in San Francisco (www.cancermetastasis.org), addressed the molecular mechanisms of cancer metastasis from the cancer microenvironment to the sentinel lymph nodes in the majority of the cases, then to systemic sites in 20 review articles and 1 commentary’ should have read ‘The 9th International Congress on Cancer Metastasis through the Lymphovascular System, being held between May 4–6, 2019, in San Francisco (www.cancermetastasisconference.org), addressed the molecular mechanisms of cancer metastasis from the cancer microenvironment to the sentinel lymph nodes in the majority of the cases, then to systemic sites in 21 review articles and 2 commentaries’.

In the paragraph starting ‘In this Special Issue: Molecular…’ and ‘Jamie Magrill, Dan Moldoveanu…’ and ‘Regarding the host immune…’ in this article, the term ‘tumor’ should have read ‘cancer’.

In the paragraph starting ‘The paper on Immune Responses…’ in this article, the sentence ‘The paper on Immune Responses against Cancer by Stanley Leong describes the background of immune interaction with cancer’ should have read ‘The paper on Immune Responses against Cancer by Stanley Leong describes the background of immune interaction with cancer, summary of the checkpoint inhibition immunotherapy trials and the diversity….’

In the sentence beginning ‘The above-described manuscripts…’ in this article, the text ‘in San Francisco (www.cancermetastasis.org) have been compiled into this Special Issue…’ should have read ‘in San Francisco (www.cancermetastasisconference.org) have been compiled into this Special Issue…’.

In the sentence beginning ‘We greatly appreciate the excellent…’ in this article, the text ' They are acknowledged on our website (www.cancermetastasis.org). We are indebted…’ should have read ' They are acknowledged on our website (www.cancermetastasisconference.org). We are indebted…’.

In the sentence beginning ‘We greatly appreciate the excellent…’ in this article, the term ‘in the Fall of 2025’ should have read ‘from October 29-31, 2025’.

In this article the title was incorrectly given as ‘Cancer metastasis through the lymphovascular system: molecular mechanisms of cancer metastasis’ but should have been ‘Editorial: Cancer metastasis through the lymphovascular system: molecular mechanisms of cancer metastasis’.

In the sentence beginning ‘David Nathanson and Ian Wood deliver…’ in this article, the text ‘a personal perspective’ should have read ‘a personal perspective as a commentary….’

The original article has been corrected.

